# Investigating Potential Correlations between Calcium Metabolism Biomarkers and Periprocedural Clinical Events in Major Cardiovascular Surgeries: An Exploratory Study

**DOI:** 10.3390/jcm13082242

**Published:** 2024-04-12

**Authors:** Adrian Ștef, Constantin Bodolea, Ioana Corina Bocșan, Ștefan Cristian Vesa, Raluca Maria Pop, Simona Sorana Cainap, Alexandru Achim, Oana Antal, Nadina Tintiuc, Anca Dana Buzoianu

**Affiliations:** 1 Clinical Department of Anesthesia and Intensive Care, Heart Institute “Niculae Stancioiu”, “Iuliu Hatieganu” University of Medicine and Pharmacy, Motilor 19-21, 400001 Cluj-Napoca, Romania; 2 Anesthesia and Intensive Care 2 Discipline, “Iuliu Hatieganu” University of Medicine and Pharmacy, Victor Babes Nr 8 Street, 400012 Cluj-Napoca, Romania; 3 Cardiology Department, Heart Institute “Niculae Stancioiu”, “Iuliu Hatieganu” University of Medicine and Pharmacy, Motilor 19-21, 400001 Cluj-Napoca, Romania; 4 Department of Pharmacology, Toxicology and Clinical Pharmacology, “Iuliu Hatieganu” University of Medicine and Pharmacy, Victor Babes Nr 8 Street, 400012 Cluj-Napoca, Romania; corinabocsan@yahoo.com (I.C.B.); raluca_parlog@yahoo.com (R.M.P.);; 5 Department of Mother and Child, 2nd Pediatric Discipline, “Iuliu Hatieganu” University of Medicine and Pharmacy, Victor Babes Nr 8 Street, 400012 Cluj-Napoca, Romania

**Keywords:** calcium, ionized calcium, vitamin D, cardiopulmonary bypass, cardiovascular surgery, open-heart surgery, intensive care

## Abstract

**Background:** There is emerging but conflicting evidence regarding the association between calcium biomarkers, more specifically ionized calcium and the prognosis of intensive care unit (ICU) postoperative cardiac patients. **Methods**: Our study investigated the relationship between ionized calcium, vitamin D, and periprocedural clinical events such as cardiac, neurologic and renal complications, major bleeding, vasoactive–inotropic score (VIS), and length of ICU and hospitalization. **Results**: Our study included 83 consecutive subjects undergoing elective major cardiac surgery requiring cardiopulmonary bypass. The mean age of the participants was 64.9 ± 8.5 years. The majority of procedures comprised isolated CABG (N = 26, 31.3%), aortic valve procedures (N = 26, 31.3%), and mitral valve procedures (N = 12, 14.5%). A difference in calcium levels across all time points (*p* < 0.001) was observed, with preoperative calcium being directly associated with intraoperative VIS (r = 0.26, *p* = 0.016). On day 1, calcium levels were inversely associated with the duration of mechanical ventilation (r = −0.30, *p* = 0.007) and the length of hospital stay (r = −0.22, *p* = 0.049). At discharge, calcium was inversely associated with length of hospital stay (r = −0.22, *p* = 0.044). All calcium levels tended to be lower in those who died during the 1-year follow-up (*p* = 0.054). Preoperative vitamin D levels were significantly higher in those who experienced AKI during hospitalization (median 17.5, IQR 14.5–17.7, versus median 15.3, IQR 15.6–20.5, *p* = 0.048) **Conclusion**: Fluctuations in calcium levels and vitamin D may be associated with the clinical course of patients undergoing cardiac surgery. In our study, hypocalcemic patients exhibited a greater severity of illness, as evidenced by elevated VIS scores, and experienced prolonged mechanical ventilation time and hospital stays. Additional larger-scale studies are required to gain a deeper understanding of their impact on cardiac performance and the process of weaning from cardiopulmonary bypass, as well as to distinguish between causal and associative relationships.

## 1. Introduction

The blood-ionized calcium concentration is strictly regulated within narrow limits by the parathyroid hormone and vitamin D. Variations in ionized calcium concentration are directly correlated with clinically significant changes in myocardial contractility, changes in blood pressure, and hemostasis [1].

For this reason, in current anesthetic practice, calcium is carefully monitored during and after a major cardiac surgery involving cardioplegia and extracorporeal circulation. Hypocalcemia and hemodynamic instability are treated with calcium salts, although there is no solid basis for this practice [2]. Moreover, reductions in ionized calcium concentrations occur at the initiation of bypass largely due to hemodilution from the priming solution [3]. Calcium shifts continue while patients are on CPB due to further hemodilution, chelation from the administration of citrated blood products, and interactions with drugs given pre- and intraoperatively [3]. Because of the critical role of calcium, normalization may be attempted before the weaning process. The rationale for calcium administration in this setting comes from the results of several small studies that showed an increase in cardiac index, stroke volume, and mean arterial pressure when this drug was administered immediately after CPB [4,5]. Contrastingly, some observational studies have shown that serum calcium concentration was positively associated with the risk of heart failure [6,7]. Calcium also was shown to have detrimental effects on myocardial cells and cardiovascular function after ischemia-reperfusion injury including cardiopulmonary bypass (CPB) [8,9]. As a result, calcium correction currently lacks clear criteria, and establishing a correlation between calcium fluctuations during surgery and clinical outcomes would amplify its significance in the operating room.

The vasoactive–inotropic score (VIS) is calculated as a weighted sum of all administered inotropes and vasoconstrictors, reflecting pharmacological support of the cardiovascular system and it predicts mortality and morbidity after cardiac surgery. Even if there is no widely accepted definition of what is “difficult weaning from CPB,” the dose of inotropes and vasopressors required during weaning is generally used for this purpose; high doses of vasoactive or inotropic agents (vasoactive–inotropic score > 10) are a mark of difficult CPB weaning and are associated with a high level of mortality during cardiac surgery [10]. Various drugs, such as catecholamines, phosphodiesterase-3 inhibitors, calcium sensitizers, and calcium salts are used to support hemodynamics during weaning from CPB. However, there is no widely accepted practice regarding the type of drug, optimal dose, and mode of drug administration, especially during weaning from CPB. Shapira et al. showed that either a bolus (10 mg/kg) or the same bolus plus the continuous infusion (1.5 mg/kg/min for 10 min) of calcium chloride (CaCl_2_) immediately after the end of CPB increased the cardiac index, stroke volume, and mean blood pressure for 3–6 min [11]. Similarly, Urban et al. demonstrated that CaCl_2_ injected at the same doses immediately after CPB weaning led to a transient increase in cardiac output and right ventricular ejection fraction [5]. In contrast, further research failed to show hemodynamic improvement as a result of CaCl_2_ administration after CPB [12,13,14].

The reason for the association between calcium levels and patient prognosis could be that calcium takes part in various physiological and biochemical processes such as heart electrophysiology and contraction, blood coagulation, neurotransmitter release, and enzyme activity regulation. These mechanisms may extend further to the entire phosphocalcium metabolism, which also includes vitamin D [15,16]. It is evident that calcium metabolism plays a crucial role in the clinical outcomes of heart surgery patients, though the precise nature and extent of its impact are yet to be fully understood. 

The primary objective of the present study was to expand the analysis of the entire spectrum of calcium metabolism along the perioperative timeframe, with the aim of exploring possible clinical correlations between these biomarkers and the outcomes of patients undergoing cardiac surgery.

## 2. Methods

### 2.1. Study Population, Blood Sampling and Follow-Up

#### 2.1.1. Design

The authors conducted a single-center, retrospective, single-arm, open-label, observational study that was approved by the “Iuliu Hatieganu” University of Medicine Ethics Committee (approval number 259 on 28 September 2023). The committee waived the need for informed consent for studies involving the use of a database.

Patients >18 years old presenting with various elective cardiovascular indications for major surgery requiring total anesthesia and cardiopulmonary bypass in the period 10 January 2021 to 28 February 2022 were included in this study. The study did not have a predefined prospective hypothesis; rather, its aim was to explore the relationship between calcium, vitamin D, and various clinical outcomes. Exclusion criteria were urgent indications and unstable patients referred for major cardiovascular surgery, patients with a history of hyperparathyroidism or active neoplasia, defective blood collection schemes, inability to collect blood repeatedly or to determine all biomarkers’ values according to the study’s protocol, incomplete information to calculate VIS score, and patients with a length of hospital length <48 h (including those who died or transferred to other hospitals). Except for these exclusion criteria, all consecutive patients were enrolled during this period. 

Vitamin D was measured 2 h before surgery. All measurements were made with the same devices, a Biotek Microplate 50 TS washer (Agilent Technologies Inc., Santa Clara, CA, USA) and a 800 TS reader (Agilent Technologies Inc., Santa Clara, CA, USA).

**Figure 1:**
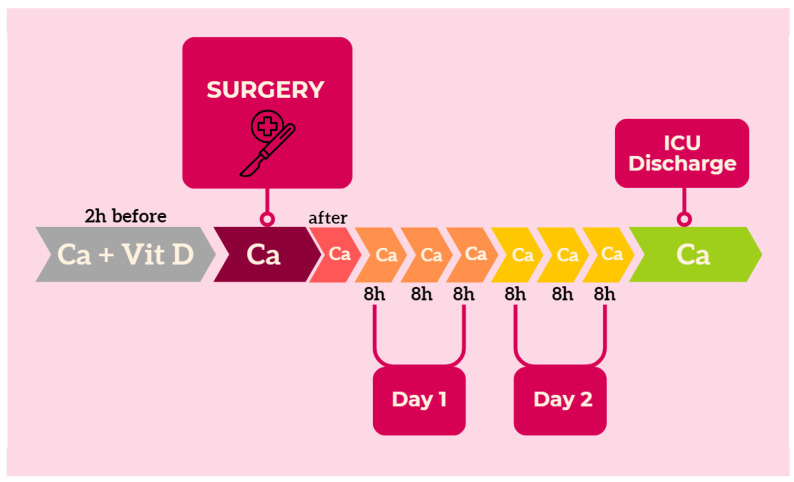
Blood sampling timeline for the study.

A timeline depicting the process of obtaining calcium biomarkers is delineated in Figure 1.

#### 2.1.2. Outcomes

All calcium and vitamin D values were correlated with the following periprocedural short-term clinical outcomes: cardiopulmonary bypass perfusion time, cross-clamp time, mechanical ventilation time, the vasoactive–inotropic score (intraoperative, at 24 h, and at 48 h), length of stay in the ICU, total length of stay in the hospital. 

Furthermore, major clinical events were recorded up to 12 months from the index procedure, including the rates of ischemic and hemorrhagic strokes, or other neurological complications, and acute kidney injury rates, and extra-cardiac hemorrhagic complications rates were measured up to discharge; all patients were followed up for up to 12 months for all-cause mortality. There was no loss of data or loss of follow-up. 

VIS was manually calculated through investigator chart review to quantify the amount of cardiovascular support required and included dopamine, dobutamine, epinephrine, milrinone, vasopressin, and norepinephrine [17]. 

### 2.2. Statistical Analyses

Continuous variables were evaluated for normality using the Shapiro–Wilk test and reported as mean ± standard deviation or median (interquartile range), as appropriate, while categorical variables were reported as frequencies and percentages. Friedman’s test and pairwise Wilcoxon’s test were used to compare repeated measurements of ionized calcium at different time points. Mann–Whitney U test was used to compare preoperative vitamin D levels and the trend in ionized calcium according to the occurrence of certain complications. Spearman’s correlation coefficients with *p*-values were calculated for the associations of preoperative vitamin D and ionized calcium with outcomes. All analyses were completed using R Statistical Software (version 4.1.1, Foundation for Statistical Computing, Vienna, Austria).

## 3. Results

### 3.1. Study Population

Baseline demographics of the 83 included patients are summarized in Table 1 along with the outcomes. 

The median CPB and aortic cross-clamp times were 100 min and 77 min, respectively. The median duration of mechanical ventilation was 3 h, while the median lengths of stay in the ICU and hospital were 3 and 9 days, respectively. The median VIS was 4 intraoperatively, 3.5 on day 0, and 1.3 on day 1. Acute kidney injury (AKI) occurred in nineteen patients (22.9%), major bleeding in two (2.4%), and an ischemic stroke in one (1.2%). A total of six patients (7.2%) died during the 1-year follow-up.

**Figure 2:**
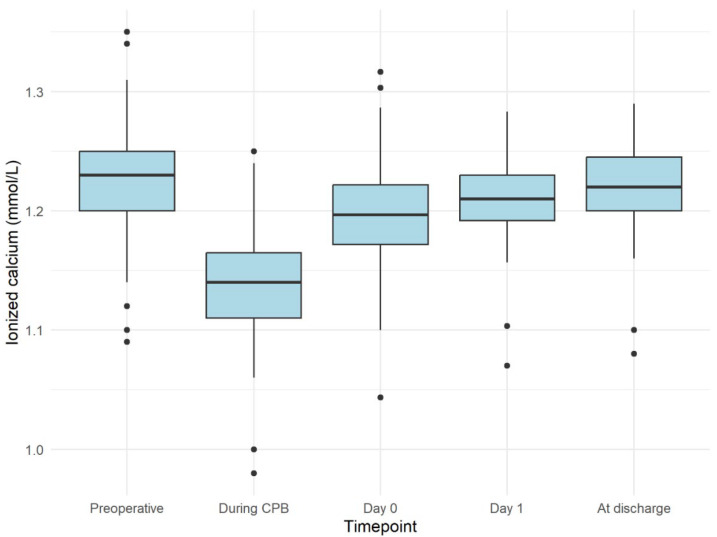
Trend in ionized calcium during the study period.

There were no significant ionized calcium alterations within the cohort, with a maximum value of 1.35 mmol/L being found before surgery and with a significant reduction during CBP, with the lowest value being 1.01 mmol/L (*p* = 0.03) (Figure 2). 

### 3.2. Preoperative Vitamin D and Trend in Ionized Calcium

The median preoperative vitamin D level was 15.8 (14.5–19.0) ng/mL. The Friedman’s test demonstrated a difference in calcium levels across all time points (*p* < 0.001), with lower levels observed during CPB (*p* < 0.001) compared to all other time points, which gradually recovered towards discharge on day 0 and day 1. The referent chosen for the analysis of calcium level differences over time was the pre-operative calcium level (calcium in mmol/L measured at admission). 

### 3.3. Association of Vitamin D and Ionized Calcium with Outcomes

Preoperative vitamin D levels were significantly higher in those who experienced AKI during hospitalization (median 17.5, IQR 14.5–17.7, versus median 15.3, IQR 15.6–20.5, *p* = 0.048), with no difference in mortality during the 1-year follow-up.

**Figure 3:**
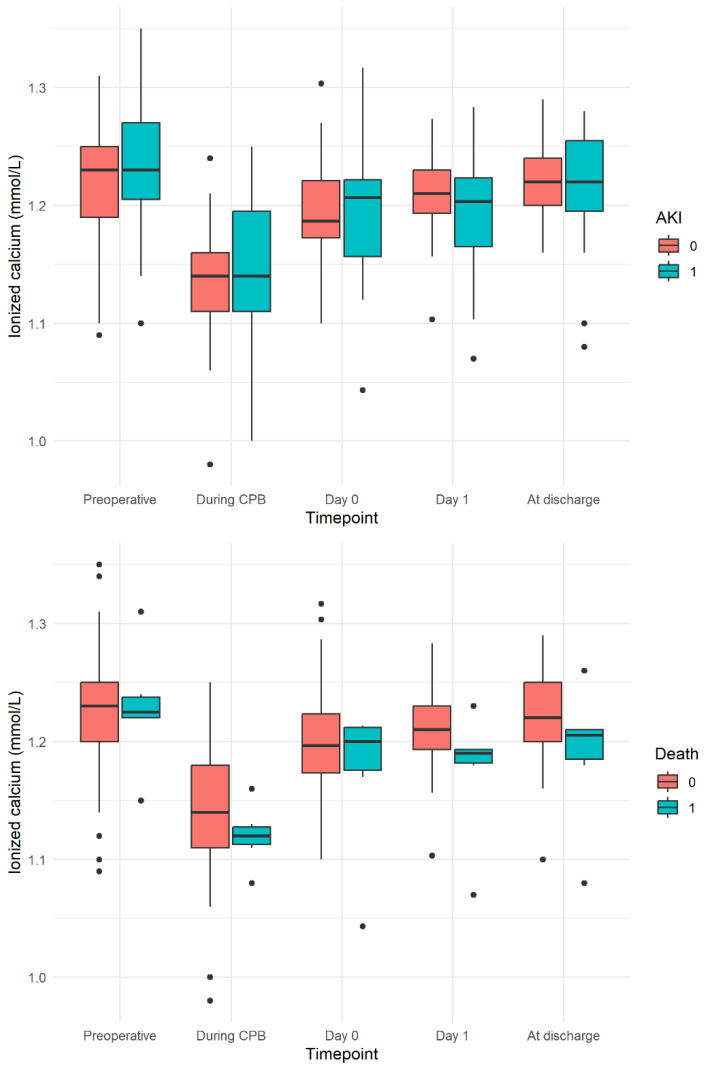
Trend in ionized calcium during the study period, according to the occurrence of a certain outcome. (**Upper panel**) AKI (0 = absent, 1 = present). (**Lower panel**) Death (0 = absent, 1 = present).

Figure 3 demonstrates the trend in ionized calcium during the study period, according to the occurrence of certain outcomes. The Mann–Whitney U test demonstrated that the overall trend in calcium did not differ according to whether a participant experienced AKI (*p* = 0.852), while calcium at all timepoints tended to be lower in those who died during the 1-year follow-up (*p* = 0.054) (Figure 3).

**Figure 4:**
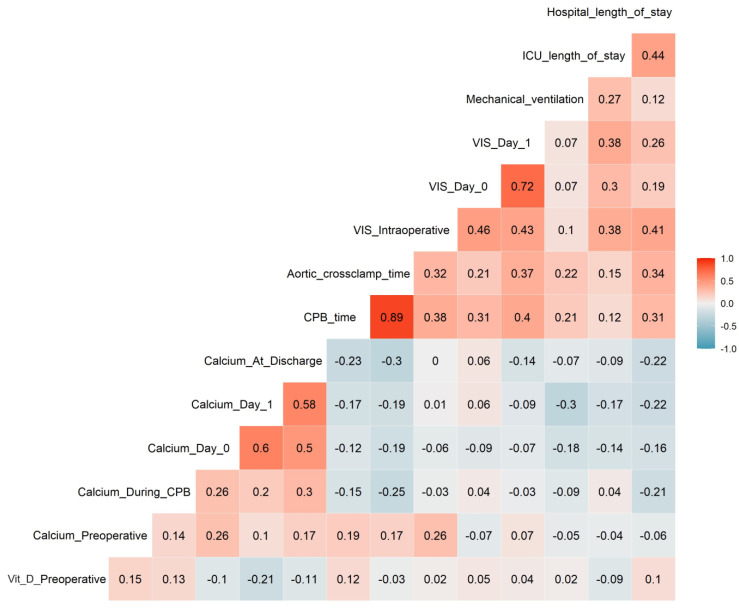
Spearman’s correlation coefficients for the associations of vitamin D and ionized calcium with continuous outcomes. Correlation matrix interpretation: each row-column pair represents a correlation of the two variables; positive correlations are displayed in red and negative correlations in blue color. Color intensity is proportional to the correlation coefficients: 0 = no correlation, −1 = perfect inverse correlation, 1 = perfect direct correlation.

**Figure 5:**
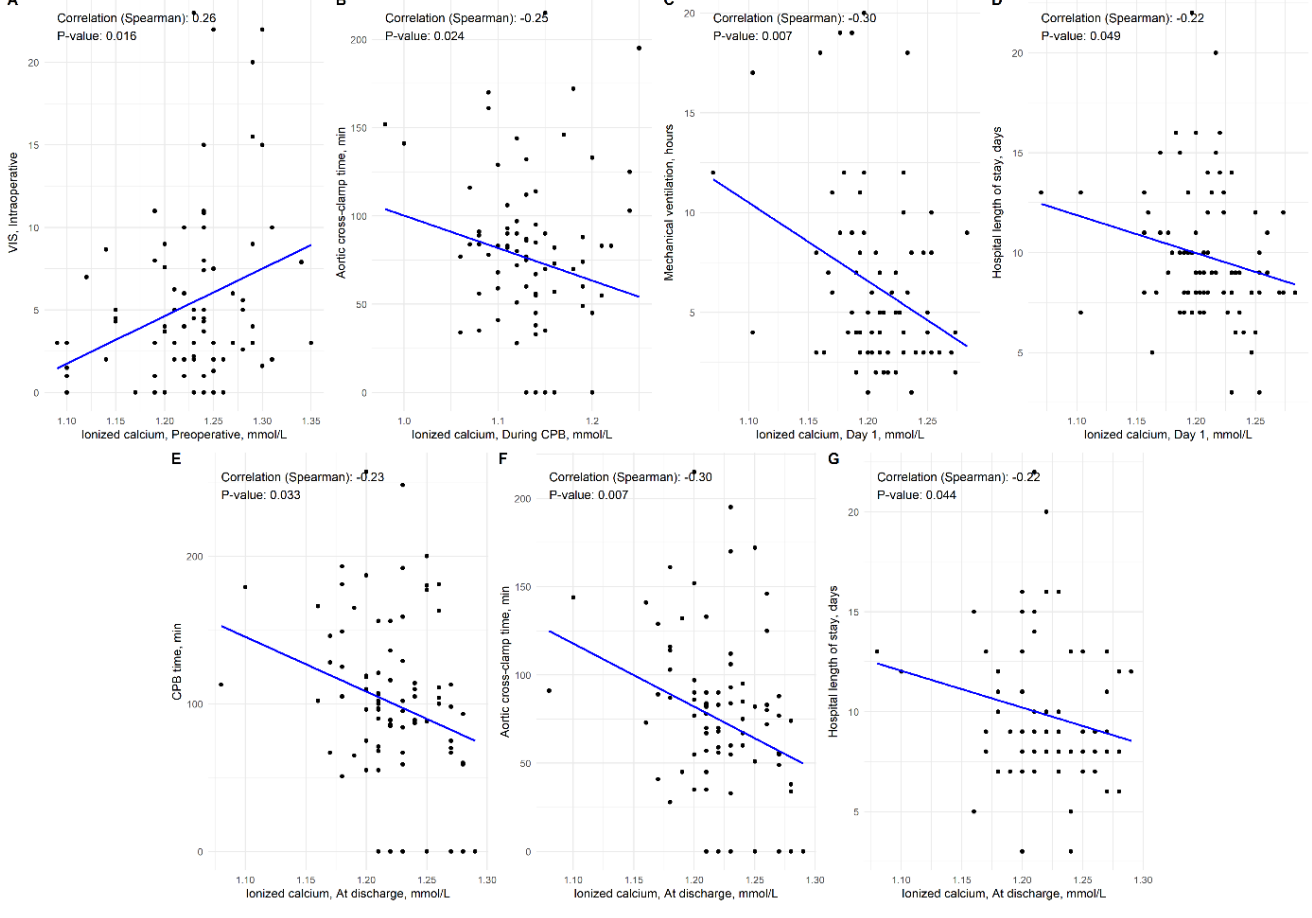
Significant correlations between ionized calcium and clinical outcomes.

Figure 4 depicts Spearman’s correlation coefficients for the associations of vitamin D and ionized calcium levels with continuous outcomes. Preoperative ionized calcium was directly associated with intraoperative VIS (r = 0.26, *p* = 0.016). The level of ionized calcium on day 1 was inversely linked with duration of mechanical ventilation (r = −0.30, *p* = 0.007) and the length of hospital stay (r = −0.22, *p* = 0.049). Finally, calcium at discharge was significantly and inversely associated with CPB time (r = −0.23, *p* = 0.033), and length of hospital stay (r = −0.22, *p* = 0.044). Significant correlations are summarized in Figure 5.

## 4. Discussion

In this study, we described the decrease in ionized calcium values during major cardiovascular surgery and how it correlates with various outcomes. The findings with a significant clinical impact were (1) intraoperative and postoperative hypocalcemia occurs frequently after cardiopulmonary bypass surgery and (2) this was significantly associated with the VIS score. Moreover, (3) ionized calcium was associated with some relevant acute parameters such as cardiopulmonary bypass time, aortic cross-clamp time, mechanical ventilation time, and length of hospital stay, (4) but also with a trend toward lower values that were associated with one-year mortality. These results deepen the importance of preoperative ionized calcium measurement. Finally, (5) vitamin D did not strongly predict postoperative outcomes but was associated with the negative evolution of AKI.

We find the robust correlation between calcium levels and the VIS score to be a key strength of our study. This connection can be elucidated by calcium’s direct influence on the heart’s chronotropic, dromotropic, and inotropic functions, as well as its role in peripheral arterial vasoregulation. While this theory is not novel [18], there is a shortage of large cohort studies in this field, with studies focusing on the correlation of calcium levels predominantly carried out within populations with ACS [19,20,21]. The most important study comes from China, from Bi et al. who studied over 6000 patients and found a significant association between worse short-term and long-term prognosis, less use of ventilation, longer ICU and hospital stays, and a higher incidence of 7-day acute kidney injury in the mild hypo-ionized calcium group (0.9–1.15 mmol/L) [22]. The authors concluded that hypocalcemia within the first day of admission to the ICU could serve as an independent prognosis factor for post-cardiovascular surgical patients [22]. Our data align with the findings of this specific study, thereby enhancing the concept of identifying and correcting calcium levels before surgery.

We also acknowledge that hypocalcemic patients may have a poorer postsurgical outcome that has an associative rather than a causative explanation, as in hypocalcaemia is a marker rather than a direct cause of disease severity. Sicker, more critical patients receive longer CBP and mechanical ventilation times and stay within the ICU for longer. At the same time, these high-risk patients often present with low levels of serum ionized calcium and vitamin D, with reported rates between 55 and 85% [23]. Calcium administration in cardiac arrest has shown no benefit and may provoke harm. In addition, no randomized controlled trial has assessed the risks and benefits of calcium supplementation in critically ill hypocalcemic patients. Several recent studies conclude that it may even harm septic ICU patients [24,25]. These observations are supported by evidence that septic patients using calcium channel blockers may have better outcomes [26,27]. There are several proposed protective mechanisms underlying this association, including the inhibition of pro-inflammatory factors, muscle proteolysis, and immunosuppression by CCB, but most importantly, the inhibition of excessive calcium influx into the cell and, as a result of this reduction in cellular toxicity, attenuation of further tissue damage and organ dysfunction [23]. On the other hand, in the largest observational study on calcium ICU supplementation published by Zhang et al., calcium supplementation was found to be independently associated with better 28-day survival [28]. Nevertheless, as the patients from our study received elective cardiac procedures, the reported rate of sepsis pre- or post-surgery was very low (3.6%). In this still unclear landscape, we hope that our results to will stimulate robust studies to overcome this limiting relationship. 

In a global survey that included 100 centers, Lomivorotov et al. found a rate of 88% of intraoperative use of calcium salts (either chloride or gluconate), especially during weaning from CPB (71%) [2]. This practice could be extended to the preoperative phase, offering potential benefits. Zhang et al. demonstrated that the level of ionized calcium at towards discharge was significantly lower in non-survivors than in survivors (1.11 ± 0.14 vs. 1.13 ± 0.10 mmol/L, *p* < 0.001), while the mild and severe hypocalcemia patients (9193 and 105 patients, respectively) did not show significant associations [29]. The same study found that mild hypercalcemia provided a protective effect against mortality, suggesting that calcium supplementation may potentially benefit critically ill patients [29]. Consistently, Choi and colleagues observed a robust link between the presence of initial hypocalcemia and mortality in a cohort of 255 consecutive trauma patients. Notably, this association persisted even after adjustment for significant confounding factors [30]. But not all studies demonstrated the predictive value of lower ionized calcium levels for a worse prognosis [25,31,32,33]. It is possible that perturbations in the ionized calcium values in acutely ill patients represent an adaptive and protective response, and some data suggest that calcium administration may be harmful in critical illness. A study showed that, even though calcium levels drop during the onset of CPB, parathormone levels markedly increase and calcium levels return almost to normal before the patient emerges from the bypass [34]. On the other hand, open-heart surgery induces acute stress and acute stress is detrimental to circulating 25-hydroxyvitamin D (25(OH)D) concentration. Low vitamin D is a global pandemic and is associated with a higher prevalence in all-cause and cardiovascular mortality. Low vitamin D levels are prevalent before cardiac surgery and are correlated with major adverse cardiac and cerebrovascular events during inpatient care and mortality months after cardiac surgery [35,36]. Examining the fluctuations in calcium and vitamin D within this subpopulation remains pertinent, despite advancements in percutaneous coronary intervention (PCI) [37,38,39], as major cardiac surgery continues to serve as a cornerstone in managing various cardiovascular disorders.

Except for certain conditions like renal failure and hypomagnesemia, disorders affecting calcium regulation are rare within the general population. Typically, they come to clinical attention due to symptoms like tetany or weakness, or within contexts where a disruption to the calcium balance is expected, such as post-parathyroid surgery, during osteoporosis diagnosis, or after cisplatin administration. As a result, the assessment of calcium levels is generally warranted when the clinical scenario heightens the likelihood of a calcium balance disorder. In contrast, disturbances in ionized calcium levels are quite common among ICU patients and are often not linked to underlying calcium regulation disorders [40,41]. Thus, in the absence of high-quality data derived from a randomized controlled trial on ionized calcium measurement and intervention, which is an urgent requirement, a broad spectrum of strategies, ranging from frequent and systematic measurement and intervention (termed a ‘liberal’ approach) to infrequent or no routine measurement and intervention (referred to as a ‘conservative’ approach), can be considered justifiable based on the limited data that are currently available [42]. The authors hope that an approach guided by rational principles, with predefined objectives for measurement, treatment, and response (potentially shifting the critical timing to the preoperative phase rather than upon ICU admission), can mitigate certain inefficiencies and the potential harm that are often associated with a strategy driven solely by the goal of normalization and the pursuit of maintaining physiological equilibrium.

There are several limitations of our study: the small sample size, being carried out in a single center, and having an observational design limited the strength and the external validity of our results. Additionally, our reliance on hospital data introduced greater variability and uncertainty in the timing and methods of sample collection and calcium supplementation than would be the case in a tightly controlled clinical trial focused on dose–response relationships. To mitigate these sources of error and explore a broader range of clinical outcomes beyond the limited scope of this report, there is a clear need for a prospective, placebo-controlled trial. Such a trial would investigate the effects of calcium supplementation and assess the practicality and safety of frequent calcium measurement and supplementation in hospitalized patients.

## 5. Conclusions

Fluctuations in ionized calcium and vitamin D may be associated with the clinical course of patients undergoing major cardiac surgery. Ionized calcium decreases during CPB were significantly and directly associated with the VIS score and other essential anesthetic parameters, as well as with a possible signal of worse longer-term outcomes such as hospital admission period and mortality after 12 months. Our study retains its exploratory nature and serves to generate hypotheses, given its limited sample size and observational design. Additionally, it is important to note that hypocalcemia might solely act as a marker of disease severity in these patients. A better understanding of their role in cardiac performance and weaning from cardiopulmonary bypass could lead to better outcomes in this population.

## Figures and Tables

**Table 1:** Population, procedure characteristics, and outcomes.

Variable	Value (N = 83)
Age (years)	64.9 ± 8.5
Males (n, %)	49 (59%)
BMI	28.4 (25.2–31.6)
Procedure	
Isolated CABG	26 (31.3%)
Aortic valve procedure	26 (31.3%)
Mitral valve procedure	12 (14.5%)
Complex valve procedure	4 (4.82%)
CABG + valve procedure	5 (6.02%)
Bentall procedure	3 (3.61%)
Aortic valve and ascending aorta repair	3 (3.61%)
Aortic aneurysm repair	2 (2.41%)
ASD correction	2 (2.41%)
Chronic kidney disease	18 (21.6%)
Mechanical ventilation, hours	5.00 (3.00–8.00)
ICU length of stay, days	3.00 (2.00–4.00)
Hospital length of stay, days	9.00 (8.00–12.0)
VIS, intraoperative	4.00 (2.00–7.55)
**Complications**	
AKI	19 (22.9%)
Sepsis (during hospitalization)	3 (3.61%)
Major bleeding	2 (2.41%)
Ischemic stroke	1 (1.20%)
Death during 1-year follow-up	6 (7.2%)

BMI, body mass index; CABG, coronary bypass arterial grafting; ASD, atrial septal defect; COPD, chronic obstructive pulmonary disease; PAD, peripheral artery disease.

## Data Availability

The data presented in this study are available on request from the corresponding author.

## Abbreviations

CBP: cardiopulmonary bypass; VIS, vasoactive inotropic score; ICU, intensive care unit; CaCl_2_, calcium chloride; AKI, acute kidney injury.
